# Long-Term Outcomes in Patients With Spontaneous Cerebellar Hemorrhage: An International Cohort Study

**DOI:** 10.1161/STROKEAHA.123.044622

**Published:** 2024-03-15

**Authors:** Jasper R. Senff, Sanjula D. Singh, Marco Pasi, Wilmar M.T. Jolink, Mark A. Rodrigues, Floris H.B.M. Schreuder, Julie Staals, Tobien Schreuder, Jules P.J. Douwes, Jelmer Talsma, Brenna N. McKaig, Christina Kourkoulis, Nirupama Yechoor, Christopher D. Anderson, Laurent Puy, Charlotte Cordonnier, Marieke J.H. Wermer, Peter M. Rothwell, Jonathan Rosand, Catharina J.M. Klijn, Rustam Al-Shahi Salman, Gabriël J.E. Rinkel, Anand Viswanathan, Joshua N. Goldstein, H. Bart Brouwers

**Affiliations:** Department of Neurology and Neurosurgery, Brain Center Rudolf Magnus, University Medical Center Utrecht, the Netherlands (J.R.S., S.D.S., J.P.J.D., J.T., G.J.E.R., H.B.B.).; Henry and Allison McCance Center for Brain Health, Massachusetts General Hospital, Boston (J.R.S., S.D.S., C.K., N.Y., C.D.A., J.R.).; Department of Emergency Medicine (B.N.M., J.N.G.), Massachusetts General Hospital Stroke Research Center, Harvard Medical School, Boston.; JPK Stroke Center (S.D.S., M.P., A.V.), Massachusetts General Hospital Stroke Research Center, Harvard Medical School, Boston.; Department of Neurology (J.R.S., S.D.S., C.K., N.Y., J.R.), Massachusetts General Hospital, Boston.; Center for Genomic Medicine (J.R.S., C.K., N.Y., C.D.A., S.D.S., J.R.), Massachusetts General Hospital, Boston.; Broad Institute, Cambridge (J.R.S., S.D.S., J.R.S., C.K., N.Y., C.D.A., J.R.).; University Lille, Inserm, CHU Lille, U1172 – LilNCog – Lille Neuroscience & Cognition, France (M.P., L.P., C.C.).; Neurology Department, University Hospital of Tours, INSERM U1253 iBrain, France (M.P.).; Department of Neurology, Isala Hospital, Zwolle, the Netherlands (W.M.T.J.).; Centre for Clinical Brain Sciences, The University of Edinburgh, United Kingdom (M.A.R., R.A.-S.S.).; Department of Neuroradiology, NHS Lothian, United Kingdom (M.A.R.).; Department of Neurology, Donders Institute for Brain Cognition & Behavior, Radboud University Medical Center, Nijmegen, the Netherlands (F.H.B.M.S., C.J.M.K.).; Department of Neurology and School for Cardiovascular Diseases (CARIM), Maastricht University Medical Center, the Netherlands (J.S.).; Department of Neurology, Zuyderland Medical Center, Heerlen, the Netherlands (T.S.).; Department of Neurology, Brigham and Women’s Hospital, Boston (C.D.A.).; Department of Neurology, LUMC, Leiden, the Netherlands (M.J.H.W.).; Wolfson Centre for Prevention of Stroke and Dementia, Nuffield Department of Clinical Neurosciences, University of Oxford, United Kingdom (P.M.R.).; Department of Neurosurgery, Elisabeth-TweeSteden Hospital, Tilburg, the Netherlands (H.B.B.).

**Keywords:** cerebellum, cohort studies, hemorrhagic stroke, recurrence, stroke, survival

## Abstract

**BACKGROUND:**

Spontaneous intracerebral hemorrhage (ICH) in the cerebellum has a poor short-term prognosis, whereas data on the long-term case fatality and recurrent vascular events are sparse. Herewith, we aimed to assess the long-term case fatality and recurrence rate of vascular events after a first cerebellar ICH.

**METHODS:**

In this international cohort study, we included patients from 10 hospitals (the United States and Europe from 1997 to 2017) aged ≥18 years with a first spontaneous cerebellar ICH who were discharged alive. Data on long-term case fatality and recurrence of vascular events (recurrent ICH [supratentoria or infratentorial], ischemic stroke, myocardial infarction, or major vascular surgery) were collected for survival analysis and absolute event rate calculation.

**RESULTS:**

We included 405 patients with cerebellar ICH (mean age [SD], 72 [13] years, 49% female). The median survival time was 67 months (interquartile range, 23–100 months), with a cumulative survival rate of 34% at 10-year follow-up (median follow-up time per center ranged: 15–80 months). In the 347 patients with data on vascular events 92 events occurred in 78 patients, after initial cerebellar ICH: 31 (8.9%) patients had a recurrent ICH (absolute event rate, 1.8 per 100 patient-years [95% CI, 1.2–2.6]), 39 (11%) had an ischemic stroke (absolute event rate, 2.3 [95% CI, 1.6–3.2]), 13 (3.7%) had a myocardial infarction (absolute event rate, 0.8 [95% CI, 0.4–1.3]), and 5 (1.4%) underwent major vascular surgery (absolute event rate, 0.3 [95% CI, 0.1–0.7]). The median time to a first vascular event during follow-up was 27 months (interquartile range, 8.7–50 months), with a cumulative hazard of 47% at 10 years.

**CONCLUSIONS:**

The long-term prognosis of patients who survive a first spontaneous cerebellar ICH is poor and comparable to that of patients who survive a first supratentorial ICH. Further identification of patients at high risk of vascular events following the initial cerebellar ICH is needed. Including patients with cerebellar ICH in randomized controlled trials on secondary prevention of patients with ICH is warranted.

Spontaneous intracerebral hemorrhage (ICH) is a devastating disease, with an incidence of 10 to 30 patients per 100 000 person per year worldwide. Cerebellar ICH accounts for ≈10% of all ICH.^1,2^ Most instances of cerebellar ICH are caused by small vessel disease related to hypertension or cerebral amyloid angiopathy.^3,4^ The in-hospital case-fatality rates vary from 25% to 40%,^5–7^ but data on the long-term outcome and risk of recurrent ICH and other cardiovascular events after a first cerebellar ICH are sparse. Few studies assessed long-term case fatality, with reported rates between 0% to 50%. All of these studies were single-center studies that included small numbers of patients (total range, n=22–114).^5,8–11^ This wide range in case-fatality rates may be explained by the varying inclusion criteria and duration of follow-up, which ranged between 0.5 and 6 years.^6^ The rates of recurrent ICH and other cardiovascular events following a first spontaneous ICH varied between 0% and 15%.^12–15^ To date, there are no large studies on the rates of recurrent stroke or other vascular events in patients with cerebellar ICH, which makes estimates unreliable.^9,16^ In addition to the existing literature on cerebellar ICH, previous studies have investigated the long-term outcome and risk of recurrent vascular events following ICH using a stratification based on location; nonlobar (including cerebellar, basal ganglia, and brainstem) versus lobar ICH. Current data suggest that patients who suffered a lobar ICH have an increased risk of recurrent ICH as compared with those with a nonlobar ICH. In nonlobar patients, most studies found a similar prevalence in the risk of recurrent hemorrhagic stroke and ischemic stroke after the initial ICH.^17–19^ However, one study showed an increased risk of ischemic events in patients after a nonlobar ICH compared with recurrent hemorrhagic events.^20^

Due to its neuroanatomical location, cerebellar ICH differs in treatment guidelines, complications, and short-term outcomes as compared with all other ICH.^6,21–23^ Given the lack of evidence on cerebellar ICH and the differences due to the neuroanatomical location of the cerebellum, in this study we aimed to assess the long-term case fatality and rates of recurrent ICH or other vascular events after a first spontaneous cerebellar ICH in a large international multi-center cohort study.

## METHODS

### Study Design and Population

We included all consecutive patients with cerebellar ICH admitted at different 10 medical centers across 2 continents, utilized as a convenience sample derived from different dedicated databases including patients over different periods in time: 1 from the United States from the period between 1997 and 2017, 6 from the Netherlands ranging from 2004 to 2017, 2 from the United Kingdom ranging from 2004 to 2017 and 1 from France ranging from 2004 to 2009 (Table S1). The data that support the findings of this study are available from the corresponding author upon reasonable request. This study was approved by all relevant institutional review boards of the included medical centers. Informed consent was obtained in line with local guidelines for each medical center.

The inclusion criteria were as follows: patients aged ≥18 years, with a first spontaneous cerebellar ICH, diagnosed with either computed tomography or magnetic resonance imaging within 48 hours of symptom onset, and who were discharged alive. The exclusion criteria were patients with an underlying macrovascular lesion as visible on computed tomography or magnetic resonance imaging (eg, arteriovenous malformation, aneurysm, cavernoma, dural fistula), neoplasm visible on computed tomography or magnetic resonance imaging, patients with hemorrhagic transformations of an ischemic stroke, or with a traumatic origin of the cerebellar ICH. If a macrovascular lesion became apparent on follow-up imaging, patients were excluded.

### Data Extraction

The data collection was performed both retrospectively and prospectively. One of the authors, S.D. Singh collected the data at every participating center together with an author from the corresponding center. Retrospective data were obtained via standardized research files in the electronic patient records. Prospective data collection from each cohort used for this study has been described in previous literature.^24–29^ Data extraction methods, including follow-up time per center, are presented in Table S1. Diagnosis and cut-off values for the obtained data were in line with local hospital guidelines. Data were obtained on the following characteristics: demographics (age, sex, ethnicity), vascular risk factors (smoking, alcohol, body mass index), modified Rankin Scale before the cerebellar ICH, medical history (hypertension, diabetes, hypercholesterolemia, ischemic stroke, transient ischemic attack, supratentorial ICH, subarachnoid hemorrhage, myocardial infarction, and atrial fibrillation/atrial flutter), medication use upon admission (anticoagulation, antiplatelets, antihypertensive medication, and lipid-lowering medication) and Glasgow Coma Scale on admission.

Treatment during hospitalization was categorized as either (1) conservative treatment or (2) surgical treatment. Conservative treatment consisted of optimal medical care with or without placement of an external ventricular drain in case of hydrocephalus. Surgical treatment was defined as hematoma evacuation, with or without craniectomy.^6,30^

Baseline ICH volume on admission was measured on computed tomography scan using Brainlab neuronavigation (Brainlab AG, Munich, Germany) or Analyse Direct 11.0 software (Mayo Clinic, Rochester, Minnesota) for volumetric analyses, or if not available, using the ABC/2 method.^31^ Magnetic resonance imaging was not performed regularly, and the obtained data were insufficient to include in further analyses.

We assessed case fatality at 30 days follow-up, 1-year follow-up, and long-term (defined as the most recent contact with a particular patient). We studied a composite end point of vascular events defined as the recurrence of any ICH (including both supratentorial and infratentorial ICH), ischemic stroke, myocardial infarction, or major vascular surgery (coronary artery bypass graft surgery, peripheral arterial disease surgery, and endo- or open major vascular treatment [eg, AAA treatment]). We assessed the rate of vascular events at 1-year after the initial cerebellar ICH and long-term follow-up.

### Data Analysis

Discrete variables were reported as counts and proportions. Continuous variables were reported as means and SD or, if nonnormally distributed, medians, and interquartile ranges. Normal distributions were assessed following Shapiro-Wilk tests. We performed survival analysis with corresponding Kaplan-Meier curves for long-term case fatality and recurrence of vascular events (for which only the first vascular event of each subtype was included for survival analysis, both fatal and nonfatal). Absolute event rates of recurrent ICH or other vascular events, including 95% CIs, were calculated using crude Poisson regression models with the number of events as outcomes. Cumulative survival and hazards of vascular events at 10 years were obtained based on the Kaplan-Meier curves. Percentages, survival, and hazard analysis are calculated and presented based on the number of patients with available data on a certain outcome. For data quality assessment, we divided the cohort into 2 equal segments (stratified by ICH date of onset), which returned no different results (data not shown). Additionally, we repeated the analysis excluding the hospital with the largest sample size (Massachusetts General Hospital), to assess the possibility of practice patterns from 1 center driving the results, which also returned no different results (data now shown). Because we only obtained baseline data on hospital admission, we refrained from performing Cox regression analysis on long-term case fatality due to the absence of data on risk factors, antiplatelet and anticoagulation use, and other secondary prevention methods following discharge, therefore, we were not able to include appropriate confounders to model these outcomes reliably. Given this limitation, we were unable to provide meaningful relative risks, and, therefore, refrained from these analyses.

The current article is written in line with the Strengthening the Reporting of Observational Studies in Epidemiology guidelines (Supplemental Material).

## RESULTS

**Figure 1.**
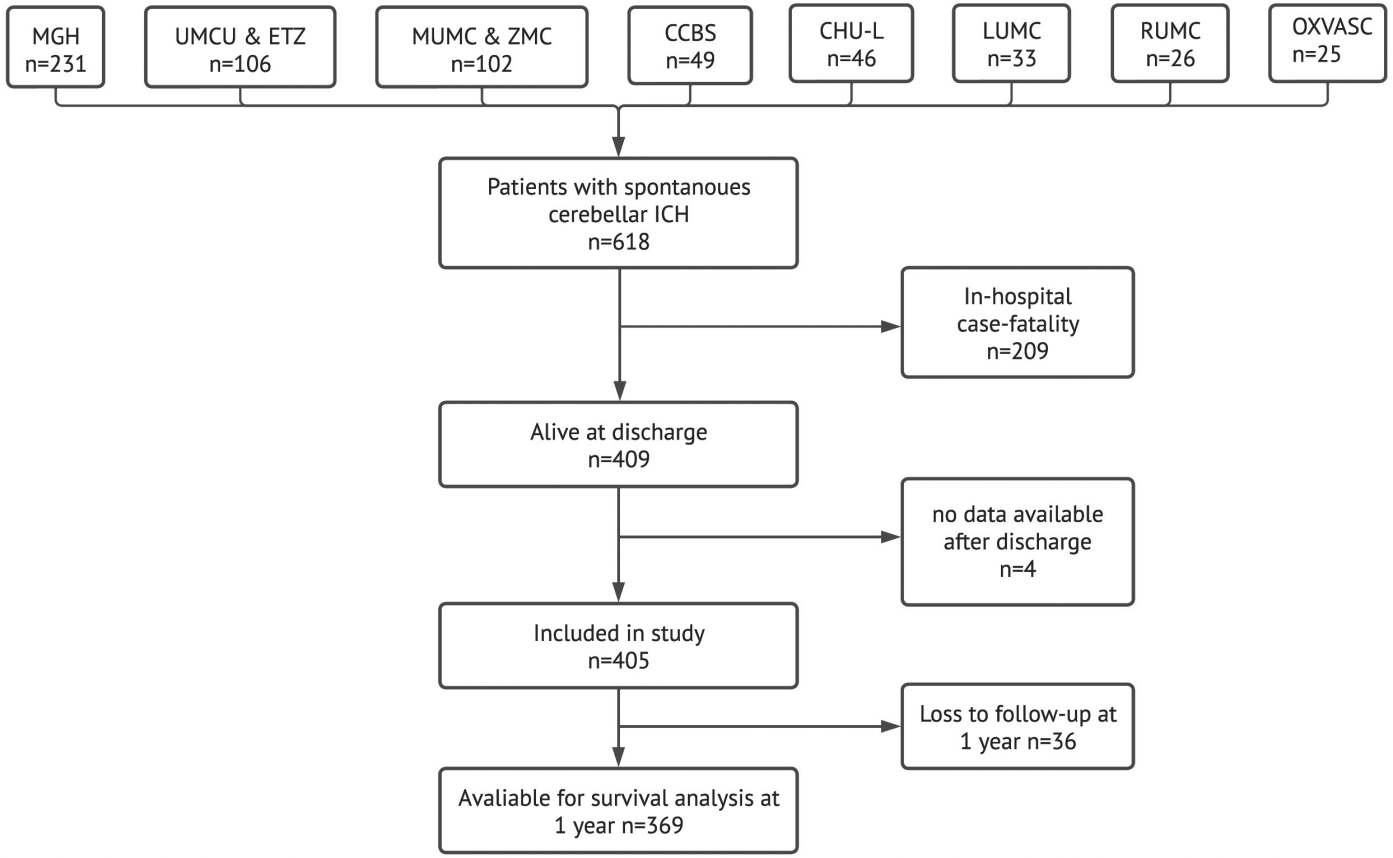
**Flowchart—inclusion per university and stratification based on in-hospital case-fatality and data availability.** n denotes the number of participants. CCBS indicates Center for Clinical Brain Science; CHU-L, Center Hospitalier Universitaire de Lille; ETZ, Elisabeth Tweesteden Ziekenhuis; ICH, intracerebral hemorrhage; LUMC, Leiden University Medical Center; MGH, Massachusetts General Hospital; MUMC, Maastricht University Medical Center; OXVASC, Oxford Vascular Study; RUMC Radboud University Medical Center; UMCU, University Medical Center Utrecht; and ZMC, Zuyderland Medisch Centrum.

During the relevant time periods at each center, 618 patients presented with spontaneous cerebellar ICH and had data available for review (Table S2). Of these 618 patients, 409 (66%) were discharged alive, of them 405 had postdischarge data available and were included in this study (Figure 1). The mean age of these 405 patients was 72 years (SD, 13), and 49% were females (Table 1). At the time of ICH occurrence, 298 of the 405 patients (74%) had a medical history of hypertension, 24% used antiplatelet therapy, and 32% used anticoagulation. Most patients were treated conservatively (71%). In addition, the baseline characteristics of this cohort of cerebellar patients with ICH stratified per center are presented in Table S3.

**Table 1.**
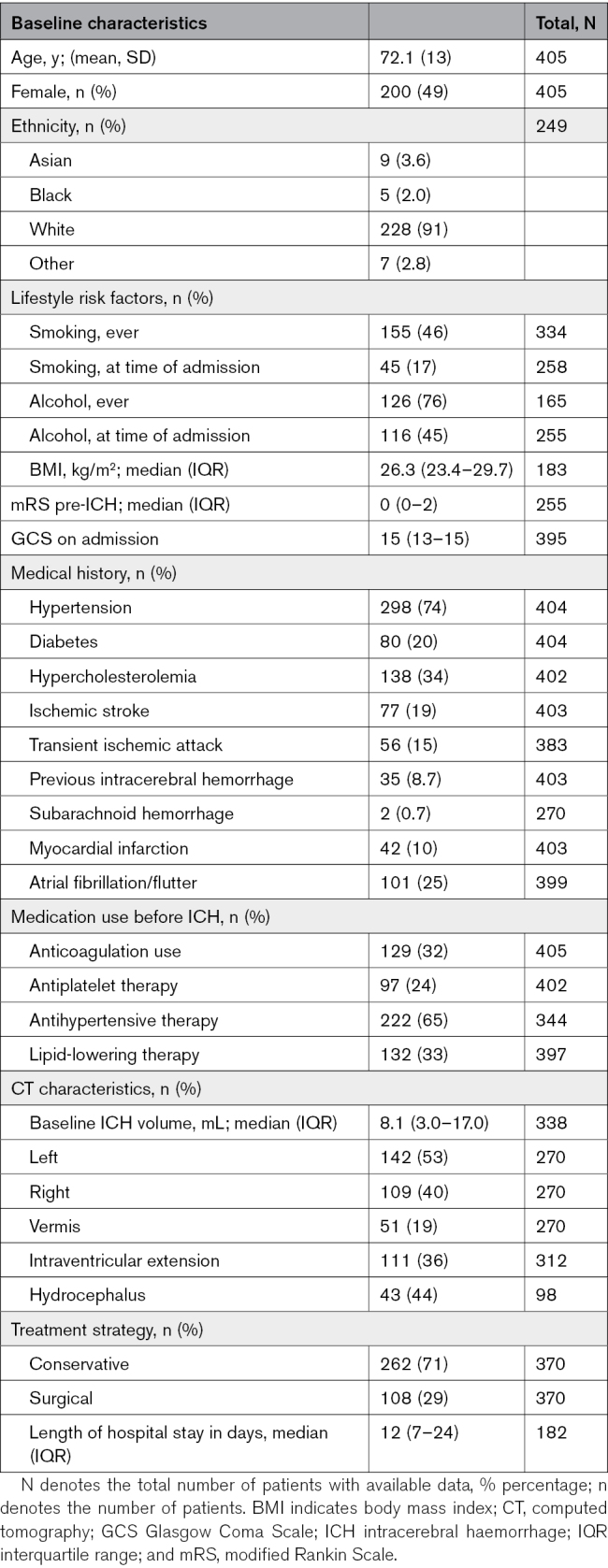
Baseline Characteristics

### Survival Analysis

**Figure 2.**
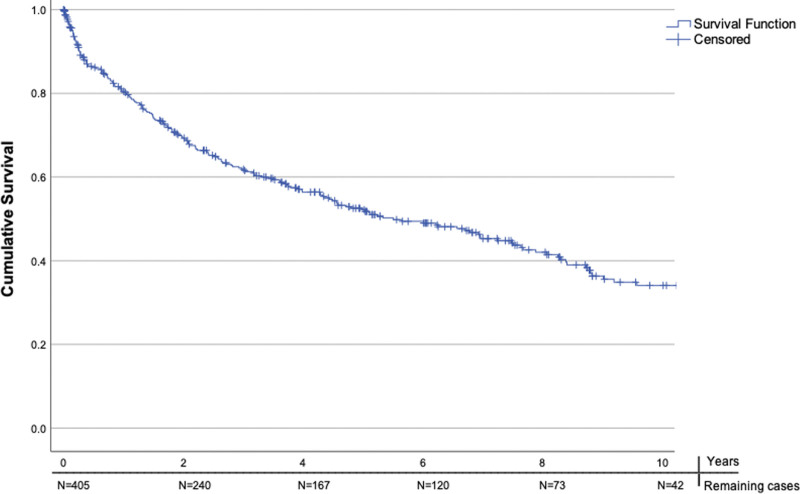
**Kaplan-Meier Curve—survival function of the patients discharged alive in years.** N denotes the number of remaining cases at a given year during follow-up.

Of the 405 patients, 28 (6.9%) died within the first 30 days after discharge. At 1-year postdischarge, 102 (25%) of the patients died. The median survival of patients discharged alive was 67.0 months (interquartile range, 23–100 months) from symptom onset. The cumulative survival rate at 10 years was 34% (Figure 2). The number of available patients for the survival analysis at the start and at 2-year intervals up to a 10-year follow-up is displayed on the *x* axis of Figure 2.

### Vascular Events: Composite Outcome

Information on vascular events after discharge was available for 347 (86%) of the included 405 patients; with a total number of patient-years of follow-up of 1711 years.

**Figure 3.**
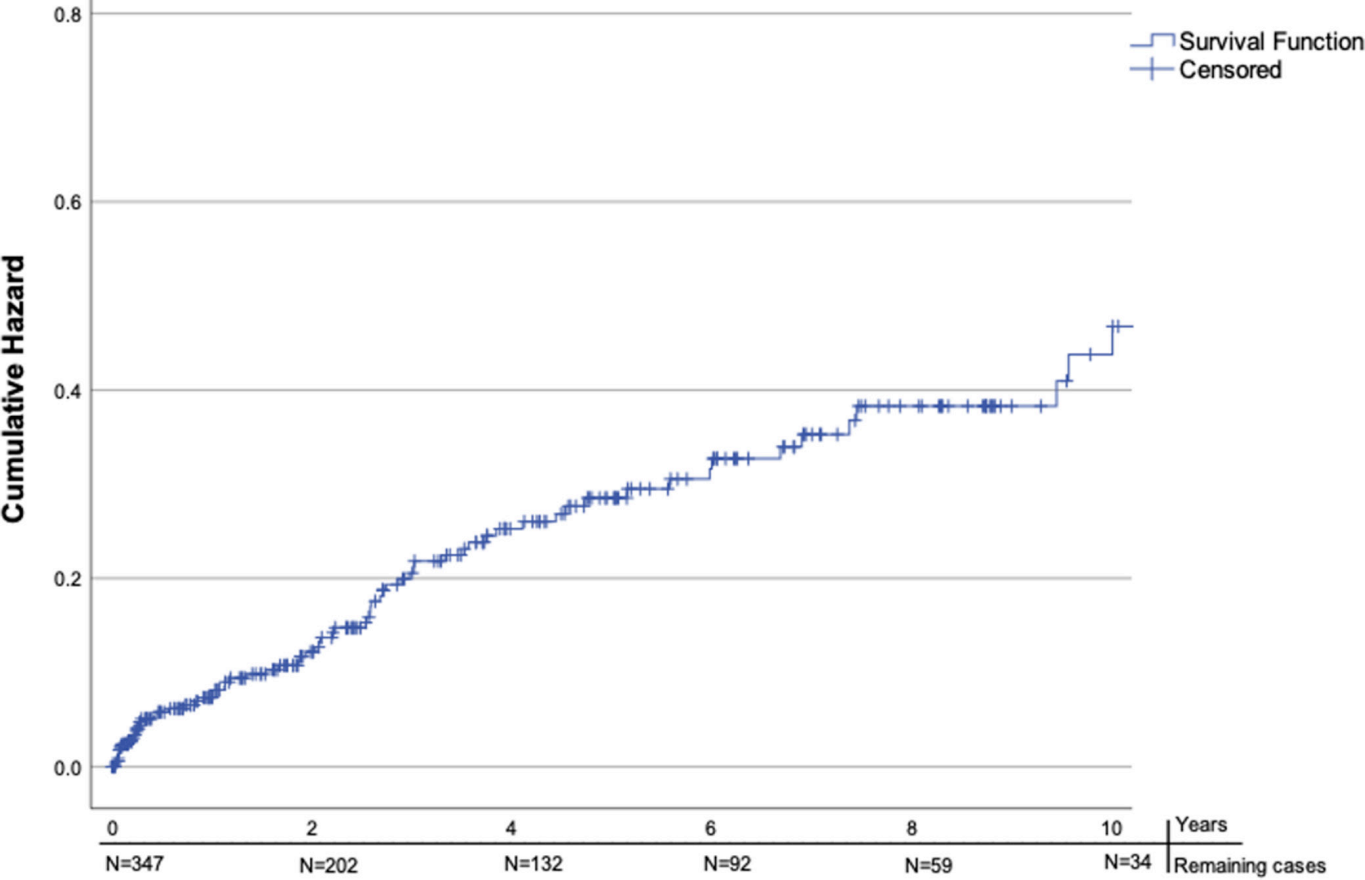
**Hazard function—cumulative hazard any first vascular event during follow-up in years.** N denotes the number of remaining cases at a given time point.

For 2 additional patients, we were only informed on the occurrence of an ischemic stroke, but data for other vascular events were not available. In total, 92 vascular events occurred in 78 (22%) patients during follow-up (Table 2). Eight patients had 2 vascular events, and 3 had 3 events during follow-up. Thirty-one individual patients (8.9%) had a recurrent ICH (supratentorial or infratentorial), 39 (11%) had an ischemic stroke, 13 (3.7%) had a myocardial infarction, and 5 (1.4%) underwent major vascular surgery. The median time to the first vascular event was 27 (interquartile range, 8.7–50) months. The absolute event rate after the cerebellar ICH was 1.8 (95% CI, 1.2–2.6) per 100 patient-years for recurrent ICH, 2.3 (95% CI, 1.6–3.2) per 100 patient-years for ischemic stroke, 0.8 (95% CI, 0.4–1.3) per 100 patient-years for myocardial infarction, and 0.3 (95% CI, 0.1–0.7) per 100 patient-years for major vascular surgery. At 1-year follow-up, 20 (7.5%) of the remaining 267 patients alive (36 patients lost to follow-up at 1 year) and available for follow-up had a first vascular event. The cumulative hazard for a first vascular event during follow-up was 47% at 10 years (Figure 3). The cumulative hazard for a recurrent ICH (both supratentorial and infratentorial) was 16%, and for ischemic stroke 23% at 10 years (Figures S1 and S2).

**Table 2.**
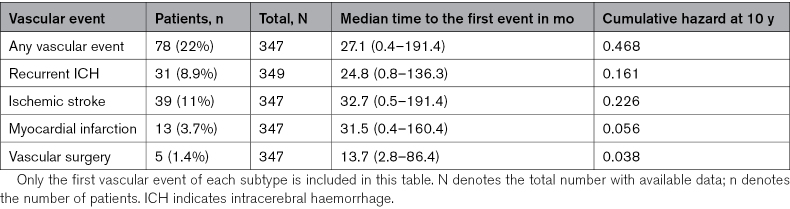
Vascular Events During Follow-Up

## DISCUSSION

Our large international cohort study has shown that one-quarter of the patients discharged alive after a first spontaneous cerebellar ICH died at 1-year follow-up. The median survival time of the patients who were discharged alive was just over 5 and a half years. Recurrent hemorrhagic and ischemic intracerebral events were found to occur with similar prevalence during follow-up.

The long-term case fatality of cerebellar ICH in our study was higher than in some previous studies.^5,9^ This may be explained by the fact that the mean age of our cohort was higher compared with these past studies and that we had significantly longer follow-up time compared with the other studies. Two studies on long-term case fatality in all spontaneous ICH, including both supratentorial and infratentorial patients with ICH alive after 7 or 30 days, respectively, show a cumulative survival at 10 years that is similar to our study.^32,33^ The rate of vascular events after initial cerebellar ICH seems similar to that in all patients with ICH.^12,14,15^ The absolute event rates of recurrent ICH, ischemic stroke, and myocardial infarction, after the initial ICH in our study are similar to those reported for nonlobar ICH in other studies.^17,18^ Previous studies describing recurrent ICH and ischemic stroke, specifically after cerebellar ICH, also found similar rates as in our study but were much smaller and therefore had wider CIs.^9,16^ We did not find other studies that assessed myocardial infarction or major vascular surgery after cerebellar ICH, and herewith presented the first data on this.

Our article has multiple strengths: first, we present the largest sample size to date on the long-term outcome of cerebellar ICH.^6,34^ Second, the data were collected at 10 medical centers across 4 countries and 2 continents, increasing the external validity of this study. Third, to secure similar data collection across centers and increase internal validity, 1 author (S.D. Singh) collected all data at every center together with a representative author of the corresponding center, following similar methods. The similar case-fatality rates upon discharge compared with previous (smaller) studies on cerebellar ICH further add to the external validity of our study.^5,7^

Limitations of this study include the retrospective data collection, with only a portion of the data being prospectively collected, and the nonstandardized follow-up times between included medical centers. The nature of data collection, including retrospective data collection from electronic patients’ files, resulted in missing data in both baseline characteristics and outcomes. Similarly, there is a notable portion of censored patients in our survival analysis due to loss to follow-up or reaching the study’s end, which could affect the estimation of the measured events. Although the study’s long-term data collection period may not fully account for secular changes in ICH outcomes, stratification at midpoint revealed consistent survival rates across the 2 periods. Additionally, the lack of information on the clinical condition at discharge and discharge destination of patients is noteworthy, as poststroke functional ability and discharge destination inform on risks of case fatality.^35^ The median age of this study was 72 years old, which is higher compared with previous studies on the long-term outcome of cerebellar ICH (median age ranging 67–68 years), which may have slightly biased our findings toward higher mortality rates.^5,9^ Furthermore, our cohort was predominantly of White ethnicity (>90%). Following past literature: if our study had incorporated a more balanced ethnical representation, we would have expected higher recurrence rates of stroke (eg, among Black and Hispanic ICH survivors).^36,37^ Our findings may also be further influenced by the cohort sourced from tertiary centers, which raises the potential for selection bias, skewing toward more severe cases. Finally, with over a third of all included patients with ICH originating from a single institution (Massachusetts General Hospital), there is a possibility of practice patterns driving the observed associations; however, similar survival and incidence rates were observed when Massachusetts General Hospital was excluded from the analysis.

The next steps for improving long-term survival and reducing the rate of recurrent ICH and other vascular events would include optimizing secondary prevention, particularly regarding antithrombotic treatment. Identifying patients at the highest risk of recurrent ICH and those at the highest risk of ischemic vascular events after the initial cerebellar ICH remains crucial. Because we refrained from performing Cox proportional hazard analyses due to a lack of collection of data points that would be regarded key confounders—a future observational study should include the following data points: (1) clinical condition at discharge and follow-up, (2) discharge destination, and (3) secondary prevention treatment post-ICH. During the follow-up of patients with cerebellar ICH, we encountered similar secondary prevention dilemmas as in patients with supratentorial ICH.^14,19^ Although antiplatelet use could be considered in the early phase after the initial ICH,^13,38^ the optimal anticoagulation strategy after an ICH still needs to be identified.^23,39,40^ Our data suggest that case-fatality rates and vascular events in cerebellar ICH are comparable to those in nonlobar supratentorial ICH, supporting the potential for further research combining cerebellar ICH and nonlobar supratentorial ICH patient groups.

The poor prognosis of patients with cerebellar ICH leaving the hospital alive with a median survival time of 5 and a half years, with over one-fifth of the patients having a vascular event during follow-up, shows that secondary prevention in those patients should be optimized. Including patients with cerebellar ICH, either alone or combined with nonlobar supratentorial patients, in randomized controlled trials on secondary prevention of patients with ICH is warranted.

## ARTICLE INFORMATION

### Sources of Funding

Dr Anderson is supported by National Institutes of Health (R01NS103924 and U01NS069673), American Heart Association (18SFRN34250007 and 21SFRN812095), and the Massachusetts General Hospital McCance Center for Brain Health for this work. The Oxford Vascular Study data derived from the Oxford Vascular Study is funded by the Welcome Trust, Wolfson Foundation, and the NIHR Biomedical Research Centre, Oxford. Floris H.B.M. Schreuder Dutch Heart Foundation (Senior Scientist Grant 2019T060). Funding for research outside the submitted work of the Netherlands Cardiovascular Research Initiative, which is supported by the Dutch Heart Foundation (CVON2015-01: CONTRAST [Collaboration for New Treatments of Acute Stroke]), and the support of the Brain Foundation Netherlands (HA2015.01.06). CONTRAST is additionally financed by the Ministry of Economic Affairs by means of the PPP Allowance made available by the Top Sector Life Sciences & Health to stimulate public-private partnerships (LSHM17016) and was funded in part through unrestricted funding by Stryker, Medtronic, and Cerenovus. Radboudumc and Erasmus MC received additional unrestricted funding on behalf of CONTRAST, for the execution of the Dutch ICH Surgery Trial pilot study and for the Dutch ICH Surgery Trial from Penumbra, Inc. Radboud University Medical Center received funding through the Promising Care funding scheme of the National Health Care Institute and ZonMw (2021038368).

### Disclosures

Dr Anderson has received sponsored research support from Bayer AG and has consulted for ApoPharma unrelated to this work. Dr Rosand reports compensation from National Football League and Takeda Development Center Americas for consultant services, unrelated to this work. The other authors report no conflicts.

### Supplemental Material

Figures S1–S2

Tables S1–S3

STROBE Checklist

## Supplementary Material


