# Supplementary material for: Long-Term Outcomes in Patients With Spontaneous Cerebellar Hemorrhage: An International Cohort Study
Source: Stroke. 2024 Mar 15;55(5):1210–7. doi: 10.1161/STROKEAHA.123.044622 (PMC11045548; doi:10.1161/STROKEAHA.123.044622)
Supplement: Supplementary file 1 [file str-55-1210-s001.pdf]

# Supplemental Material to: Long-Term Outcomes in Patients with Spontaneous Cerebellar Hemorrhage: An International Cohort Study

**Figure S1.** Hazard function – cumulative hazard new intracerebral hemorrhage during follow-up

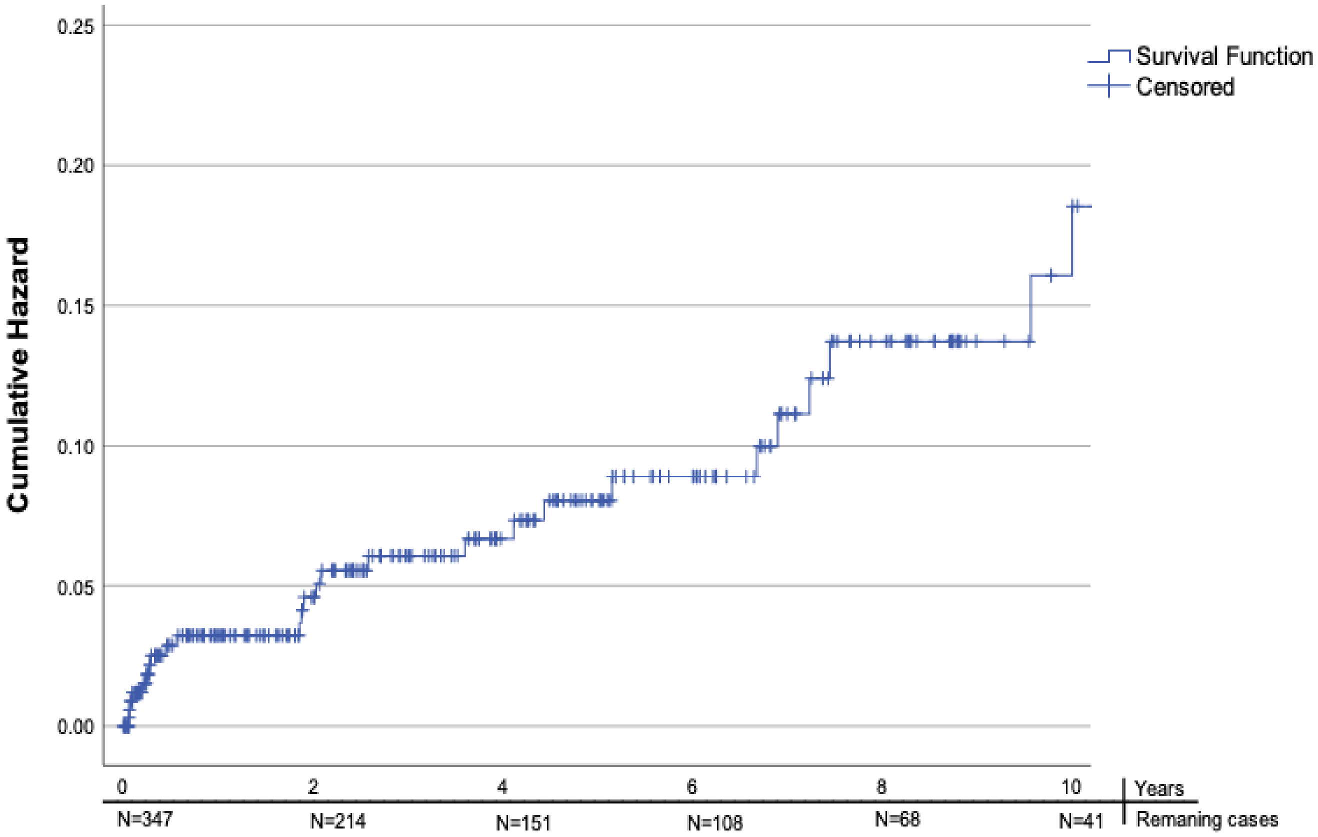


Legend S1:
Abbreviations: N denotes the number of remaining cases at a given time point.

**Figure S2.** Hazard function – cumulative hazard ischemic stroke during follow-up

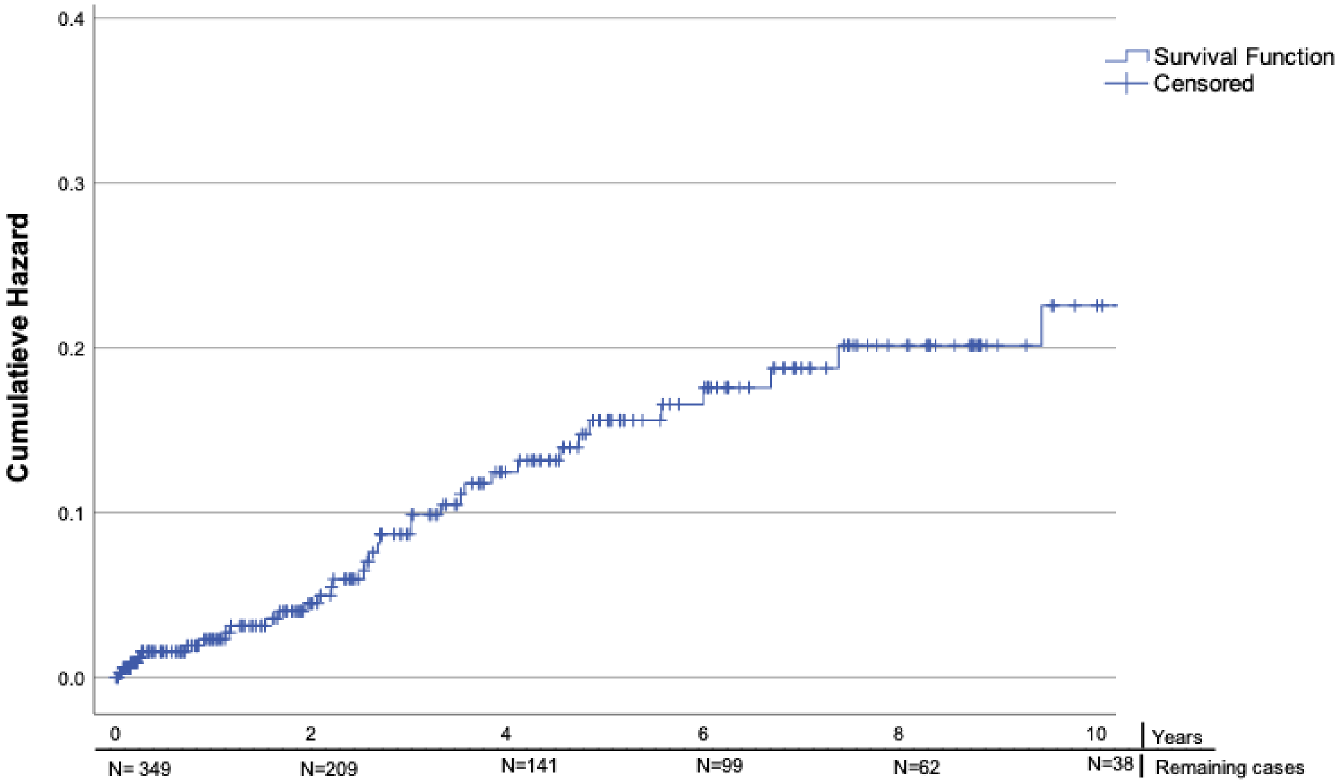


Legend S2.
Abbreviations: N denotes the number of remaining cases at a given time point.

**Table S1 –** Data collection, extraction and follow-up time per center

| Center | Location | Period | Data collection and extraction | Median follow-up time months (IQR) |
|---|---|---|---|---|
| Massachusetts General Hospital (MGH) | Boston, United States of America | 1997-2017 | Retrospective database from standardized research records in electronic patient files combined with most recent contact in clinical and research records, death certificates.<br>Prospective: MGH ICH Cohort[24] | 40 (15.8-91.4) |
| University Medical Center Utrecht (UMCU) + Elizabeth TweeSteden Ziekenhuis (ETZ) | Utrecht & Tilburg, The Netherlands | 2007-2016 | Retrospective database from standardized research records in electronic patient files combined with most recent contact in clinical and research records, death certificates, and phone calls to general practioners.<br>Prospective: The Parelsnoer Initiative[25] | 35 (4.2-69) |
| Maastricht University Medical Center (MUMC) + Zuyderland Medisch Centrum (ZMC) | Maastricht & Heerlen/Sittard, The Netherlands | 2004-2009 | Retrospective database from standardized research records in electronic patient files combined with most recent contact in clinical and research records, death certificates. | 21 (5.8-91) |
| Centre Hospitalier Universitaire de Lille (CHU-L) | Lille, France | 2004-2009 | Prospective: Prognosis of InTraCerebral Haemorrhage (PITCH) Cohort[26] | 80 (13-122) |
| Leiden University Medical Center (LUMC) | Leiden, The Netherlands | 2010-2018 | Retrospectively: most recent contact in clinical and research records, death certificates | 15 (0.9-29) |
| Radboud University Medical Center (RUMC) | Nijmegen, The Netherlands | 2013-2017 | Retrospective database from standardized research records in electronic patient files combined with most recent contact in clinical and research records, death certificates. | 46 (31-64) |
| John Radcliffe Hospital University of Oxford (OXVASC) | Oxford, United Kingdom | 2004-2017 | Prospective: Oxford Vascular Study (OXVASC)[27] | 63 (44-106) |
| Clinical Brain Science Universy of Edinburgh (CCBS) | Edinburgh, United Kingdom | 2010-2016 | Prospective: The Lothian Audit of the Treatment of Cerebral Hemorrhage (LATCH)[28] and (Lothian IntraCerebral Hemorrhage, Pathology, Imaging and Neurological Outcome) LINCHPIN[29] | 44 (14-74) |

Legend Table S1. Data collection, extraction and follow-up time per center. Abbrevations: IQR: Interquartile Range

**Table S2** – Baseline characteristics, full cohort, including non-survivors.

| **Baseline characteristics** | **Full cohort, including non-survivors** (n=618) |
|---|---|
| Age in years, (mean, SD) | 73.0 (13) |
| Female, n (%) | 307 (50) |
| Ethnicity, n (%) | |
| Asian | 15 (4.3) |
| Black | 8 (2.3) |
| White | 319 (91) |
| Other | 9 (2.6) |
| Lifestyle risk factors, n (%) | |
| Smoking, ever | 218 (46) |
| Smoking, at time of admission | 70 (11) |
| Alcohol, ever | 167 (73) |
| Alcohol, at time of admission | 147 (45) |
| BMI in kg/$m^2$, median (IQR) | 26.3 (23.4-29.4) |
| mRS pre-ICH, median (IQR) | 0 (0-2) |
| GCS on admission, median (IQR) | 14 (8-15) |
| Medical history, n (%) | |
| Hypertension | 451 (74) |
| Diabetes Mellitus | 111 (18) |
| Hypercholesterolemia | 208 (34) |
| Ischemic Stroke | 107 (18) |
| Transient Ischemic Attack | 79 (14) |
| Previous intracerebral hemorrhage | 44 (7.2) |
| Subarachnoid hemorrhage | 2 (0.5) |
| Myocardial infarction | 66 (11) |
| Atrial fibrillation/flutter | 161 (27) |
| Medication use prior to ICH, n (%) | |
| Anticoagulation use | 210 (31) |
| Antiplatelet therapy | 139 (23) |
| Antihypertensive therapy | 331 (65) |
| Lipid-lowering therapy | 182 (31) |
| CT characteristics, n (%) | |
| Baseline ICH volume in mL, median (IQR) | 13.0 (4.4-26.8) |
| Left | 191 (49) |
| Right | 168 (43) |
| Vermis | 89 (23) |
| Intraventricular extension | 224 (49) |
| Hydrocephalus | 71 (54) |
| Treatment strategy, n (%) | |
| Conservative | 406 (66) |
| Surgical | 159 (28) |
| Length of hospital stay in days, median (IQR) | 9 (3-19) |

Legend Table S2: *Abbreviations: n denotes the total number of participants, % percentage, IQR inter-quartile range, SD standard deviation, BMI body mass index, mRS modified Rankin Scale, ICH intracerebral hemorrhage, GCS Glasgow Coma Scale, SBP systolic Blood Pressure and DBP Diastolic Blood Pressure, EVD External Ventricular Drainage, LoS Length of Stay*

**Table S3 –** baseline characteristics of included patients per center

| **Baseline characteristics included patients** | **MGH** (n=153) | **UMCU & ETZ** (n=84) | **MUMC & ZMC** (n=59) | **CCBS** (n=32) | **CHU-L** (n=18) | **LUMC** (n=26) | **RUMC** (n=14) | **OXVASC** (n=19) |
|---|---|---|---|---|---|---|---|---|
| Age in years, (mean, SD) | 72.7 (13.5) | 69.5 (12.0) | 74.9 (11.7) | 74.6 (15.3) | 67.7 (14.2) | 72.2 (9.2) | 67.9 (14.9) | 73.8 (15.1) |
| Female, n (%) | 71 (46.4) | 43 (51.2%) | 38 (64.4) | 13 (40.6) | 9 (50.0) | 12 (46.2%) | 5 (35.7) | 11 (57.9) |
| Ethnicity, n (%) | | | | | | | | |
| Asian | 9 (6.2) | 0 (0) | NA | NA | NA | 0 | 0 (0) | 0 (0) |
| Black | 2 (1.4) | 2 (4.4) | NA | NA | NA | 0 | 0 (0) | 1 (5.3) |
| White | 134 (92.2) | 42 (91.3) | NA | NA | NA | 18 (94.7) | 14 (100) | 18 (94.7) |
| Other | 4 (2.8) | 2 (4.4) | NA | NA | NA | 1 (5.3) | 0 (0) | 0 (0) |
| Lifestyle risk factors, n (%) | | | | | | | | |
| Smoking, ever | 69 (55.2) | 40 (57.1) | 12 (30.8) | 15 (46.9) | 2 (11.1) | 11 (52.4) | 4 (36.43) | 2 (11.1) |
| Smoking, at time of admission | 15 (12) | 17 (24.6) | NA | 7 (21.9) | NA | 3 (13.6) | 3 (27.3) | NA |
| Alcohol, ever | 49 (100) | 35 (70) | NA | 11 (100) | 3 (16.7) | 17 (85.0) | 4 (40.0) | 7 (100) |
| Alcohol, at time of admission | 49 (40.5) | 31 (50) | NA | 11 (37.9) | NA | 14 (66.7) | 4 (40.0) | 7 (58.3) |
| BMI in kg/m$^2$, median (IQR) | 27.0 (24.4-30.9) | 25.7 (22.6-28.0) | NA | NA | NA | 27.4 (23.3-31.7) | 23.4 (23.0-26.3) | NA |
| mRS pre-ICH, median (IQR) | 1 (0-2) | 0 (0-2) | NA | 1 (1-3) | 0 (0-1) | 0 (0-2) | 0 (0-2) | NA |
| GCS on admission, median (IQR) | 15 (14-15) | 14 (12-14) | 15 (13-15) | 14 (14-15) | 15 (14-15) | 15 (14-15) | 14 (13-15) | 15 (14-15) |
| Medical history, n (%) | | | | | | | | |
| Hypertension | 128 (84.2) | 54 (64.3) | 41 (69.5) | 25 (78.1) | 13 (72.2) | 15 (57.7) | 8 (57.2) | 14 (73.7) |
| Diabetes Mellitus | 37 (24.3) | 12 (14.3) | 13 (22.0) | 3 (9.4) | 5 (27.8) | 7 (26.9) | 0 (0) | 3 (15.8) |
| Hypercholesterolemia | 74 (48.7) | 20 (24.4) | 16 (28.1) | 6 (18.8) | 5 (27.8) | 7 (26.9) | 4 (28.6) | 6 (31.6) |
| Ischemic Stroke | 38 (25.0) | 7 (8.3) | 10 (17.0) | 10 (31.3) | 1 (5.6) | 6 (24.0) | 1 (7.1) | 4 (21.1) |
| Transient Ischemic Attack | 16 (12.1) | 12 (14.3) | 9 (15.3) | 6 (18.8) | 2 (11.1) | 6 (24.0) | 2 (14.3) | 3 (15.8) |
| Previous intracerebral hemorrhage | 15 (10.0) | 5 (6.0) | 6 (10.2) | 3 (9.4) | 0 (0) | 5 (19.2) | 1 (7.1) | 0 (0) |
| Subarachnoid hemorrhage | 2 (1.4) | 0 (0) | NA | NA | NA | 0 (0) | 0 (0) | NA |
| Myocardial infarction | 9 (5.9) | 5 (6.0) | 18 (30.5) | 8 (25.0) | 1 (5.6) | 0 (0) | 1 (7.1) | 0 (0) |
| Atrial fibrillation/flutter | 39 (26.2) | 13 (15.5) | 15 (26.3) | 16 (50.0) | 1 (5.6) | 10 (38.5) | 2 (14.3) | 5 (26.3) |

Legend Table S3:
Abbreviations: MGH Massachusetts General Hospital, UMCU University Medical Center Utrecht, ETZ Elisabeth Tweesteden Ziekenhuis, MUMC Maastricht University Medical Center, ZMC Zuyderland Medisch Centrum, CCBS Center for Clinical Brain Science, University of Edinburgh, CHU-L Centre Hospitalier Universitaire de Lille, LUMC Leiden University Medical Center, OXVASC The Oxford Vascular Study, University of Oxford, RUMC Radboud University Medical Center, Nijmegen, n denotes the total number of participants, % percentage, IQR inter-quartile range, SD standard deviation, BMI body mass index, mRS modified Rankin Scale, ICH intracerebral hemorrhage, GCS Glasgow Coma Scale, mL milliliter, NA not applicable.

| **Baseline characteristics included cohort** | **MGH** (n=153) | **UMCU & ETZ** (n=84) | **MUMC & ZMC** (n=59) | **CCBS** (n=32) | **CHU-L** (n=18) | **LUMC** (n-26) | **RUMC** (n=14) | **OXVASC** (n=19) |
|---|---|---|---|---|---|---|---|---|
| Medication use prior to ICH, n (%) | | | | | | | | |
| Anticoagulation use | 42 (27.5) | 31 (36.9) | 21 (35.6) | 11 (34.4) | 4 (22.2) | 14 (53.9) | 3 (21.4) | 3 (15.8) |
| Antiplatelet therapy | 15 (9.9) | 28 (33.3) | 21 (35.6) | 13 (40.6) | 3 (16.7) | 8 (32.0) | 5 (35.7) | 4 (21.1) |
| Antihypertensive therapy | 103 (68.2) | 50 (59.5) | NA | 24 (75.0) | 13 (72.2) | 14 (53.9) | 7 (50.0) | 11 (57.9) |
| Lipid-lowering therapy | 63 (42.0) | 22 (26.5) | 11 (19.0) | 12 (38.7) | 4 (22.2) | 11 (45.8) | 5 (35.7) | 4 (21.1) |
| CT characteristics. n (%) | | | | | | | | |
| Baseline ICH volume in mL, median (IQR) | 7.0 (2.14-15.0) | 11.7 (5.1-19.3) | 7.2 (3.4-16.4) | 5.9 (1.8-13.4) | 11.9 (2.7-14.7) | NA | 16.6 (5.7-24.9) | NA |
| Left | 75 (49.0) | 48 (57.8) | NA | NA | NA | 8 (40.0) | 11 (78.6) | NA |
| Right | 55 (36.0) | 38 (45.8) | NA | NA | NA | 10 (50.0) | 6 (42.9) | NA |
| Vermis | 23 (15.0) | 22 (27.2) | NA | NA | NA | 3 (15.0) | 3 (21.4) | NA |
| Intraventricular extension | 44 (31.7) | 37 (44.1) | NA | 13 (40.6) | 6 (33.3) | 6 (23.1) | 5 (35.7) | NA |
| Hydrocephalus | NA | 37 (44.1) | NA | NA | NA | NA | 6 (42.9) | NA |
| Treatment Strategy, n (%) | | | | | | | | |
| Conservative | 99 (65.1) | 58 (69.0) | 41 (69.5) | NA | 14 (77.8) | 23 (95.8) | 9 (74.3) | 18 (94.7) |
| Surgical | 53 (34.9) | 26 (31.0) | 18 (30.5) | NA | 4 (22.2) | 1 (4.2) | 5 (35.7) | 1 (5.3) |
| Length of hospital stay in days, median (IQR) | NA | 14 (8-25) | 14 (8-29) | NA | NA | 9 (6-13) | 13 (6-22) | NA |

Legend Table S3:
Abbreviations: MGH Massachusetts General Hospital, UMCU University Medical Center Utrecht, ETZ Elisabeth Tweesteden Ziekenhuis, MUMC Maastricht University Medical Center, ZMC Zuyderland Medisch Centrum, CCBS Center for Clinical Brain Science, University of Edinburgh, CHU-L Centre Hospitalier Universitaire de Lille, LUMC Leiden University Medical Center, OXVASC The Oxford Vascular Study, University of Oxford, RUMC Radboud University Medical Center, Nijmegen, n denotes the total number of participants, % percentage, IQR inter-quartile range, SD standard deviation, BMI body mass index, mRS modified Rankin Scale, ICH intracerebral hemorrhage, GCS Glasgow Coma Scale, mL milliliter, NA not applicable.

STROBE Statement—checklist of items that should be included in reports of observational studies
Article title: LONG-TERM CASE-FATALITY AND RECURRENCE RATE OF VASCULAR EVENTS IN PATIENTS WITH SPONTANEOUS CEREBELLAR HEMORRHAGE: AN INTERNATIONAL COHORT STUDY

| | Item No | Recommendation | Page No |
|---|---|---|---|
| **Title and abstract** | 1 | (*a*) Indicate the study's design with a commonly used term in the title or the abstract | P1,P4 |
| | | (*b*) Provide in the abstract an informative and balanced summary of what was done and what was found | P4,5 |
| **Introduction** | | | |
| Background/rationale | 2 | Explain the scientific background and rationale for the investigation being reported | P6-7 |
| Objectives | 3 | State specific objectives, including any prespecified hypotheses | P7 |
| **Methods** | | | |
| Study design | 4 | Present key elements of study design early in the paper | P8, Table S1 |
| Setting | 5 | Describe the setting, locations, and relevant dates, including periods of recruitment, exposure, follow-up, and data collection | P8-10 Table S1 |
| Participants | 6 | (*a*) *Cohort study*—Give the eligibility criteria, and the sources and methods of selection of participants. Describe methods of follow-up<br>*Case-control study*—Give the eligibility criteria, and the sources and methods of case ascertainment and control selection. Give the rationale for the choice of cases and controls<br>*Cross-sectional study*—Give the eligibility criteria, and the sources and methods of selection of participants | P8-10 Table S1 |
| | | (*b*) *Cohort study*—For matched studies, give matching criteria and number of exposed and unexposed<br>*Case-control study*—For matched studies, give matching criteria and the number of controls per case | |
| Variables | 7 | Clearly define all outcomes, exposures, predictors, potential confounders, and effect modifiers. Give diagnostic criteria, if applicable | P 8-10 |
| Data sources/ measurement | 8* | For each variable of interest, give sources of data and details of methods of assessment (measurement). Describe comparability of assessment methods if there is more than one group | P8-10 Table S1,S3 |
| Bias | 9 | Describe any efforts to address potential sources of bias | P8-10,15,16 |
| Study size | 10 | Explain how the study size was arrived at | P8,12, Fig 1 |
| Quantitative variables | 11 | Explain how quantitative variables were handled in the analyses. If applicable, describe which groupings were chosen and why | P 9-10 |
| Statistical methods | 12 | (*a*) Describe all statistical methods, including those used to control for confounding | P 10 |
| | | (*b*) Describe any methods used to examine subgroups and interactions | P 9-10 Table S3 |
| | | (*c*) Explain how missing data were addressed | P9-10 Table S3 |
| | | (*d*) *Cohort study*—If applicable, explain how loss to follow-up was addressed<br>*Case-control study*—If applicable, explain how matching of cases and controls was addressed<br>*Cross-sectional study*—If applicable, describe analytical methods taking account of sampling strategy | P9-10,12,13 Fig 1-3, Fig S1,2 |
| | | (*e*) Describe any sensitivity analyses | |

Continued on next page

Supplemental material

| Results | | | Page |
|---|---|---|---|
| Participants | 13* | (a) Report numbers of individuals at each stage of study—eg numbers potentially eligible, examined for eligibility, confirmed eligible, included in the study, completing follow-up, and analysed | P12-13, Fig 1-3 Fig S1,S2 |
| | | (b) Give reasons for non-participation at each stage | P12,Fig 1 |
| | | (c) Consider use of a flow diagram | P12 Fig 1 |
| Descriptive data | 14* | (a) Give characteristics of study participants (eg demographic, clinical, social) and information on exposures and potential confounders | P 12 Table 1, Table S2,3 |
| | | (b) Indicate number of participants with missing data for each variable of interest | P 12 Table S3 |
| | | (c) *Cohort study*—Summarise follow-up time (eg, average and total amount) | P 8,9 Table S1 |
| Outcome data | 15* | *Cohort study*—Report numbers of outcome events or summary measures over time | P12-13 Fig 2,3 Fig S1,S2 |
| | | *Case-control study*—Report numbers in each exposure category, or summary measures of exposure | |
| | | *Cross-sectional study*—Report numbers of outcome events or summary measures | |
| Main results | 16 | (*a*) Give unadjusted estimates and, if applicable, confounder-adjusted estimates and their precision (eg, 95% confidence interval). Make clear which confounders were adjusted for and why they were included | P12, Fig 2 |
| | | (*b*) Report category boundaries when continuous variables were categorized | |
| | | (*c*) If relevant, consider translating estimates of relative risk into absolute risk for a meaningful time period | |
| Other analyses | 17 | Report other analyses done—eg analyses of subgroups and interactions, and sensitivity analyses | P12-13, Fig 1-3 Fig S1,S2 Table 2 |
| **Discussion** | | | |
| Key results | 18 | Summarise key results with reference to study objectives | P14,16 |
| Limitations | 19 | Discuss limitations of the study, taking into account sources of potential bias or imprecision. Discuss both direction and magnitude of any potential bias | P15 |
| Interpretation | 20 | Give a cautious overall interpretation of results considering objectives, limitations, multiplicity of analyses, results from similar studies, and other relevant evidence | P14-16 |
| Generalisability | 21 | Discuss the generalisability (external validity) of the study results | P 14-16 |
| **Other information** | | | |
| Funding | 22 | Give the source of funding and the role of the funders for the present study and, if applicable, for the original study on which the present article is based | P17 |

*Give information separately for cases and controls in case-control studies and, if applicable, for exposed and unexposed groups in cohort and cross-sectional studies.

**Note:** An Explanation and Elaboration article discusses each checklist item and gives methodological background and published examples of transparent reporting. The STROBE checklist is best used in conjunction with this article (freely available on the Web sites of PLoS Medicine at http://www.plosmedicine.org/, Annals of Internal Medicine at http://www.annals.org/, and Epidemiology at http://www.epidem.com/). Information on the STROBE Initiative is available at www.strobe-statement.org.
